# Smartphone dependency and mental health among Chinese rural adolescents: the mediating role of cognitive failure and parent–child relationship

**DOI:** 10.3389/fpsyg.2023.1194939

**Published:** 2023-10-05

**Authors:** Lilan Chen

**Affiliations:** ^1^School of Psychology, Hainan Normal University, Haikou, China; ^2^Adolescent Psychological Development and Education Center of Hainan, Haikou, China

**Keywords:** smartphone dependency, mental health, parent-child relationship, cognitive failure, Chinese rural adolescents

## Abstract

**Background:**

With the widespread use of smartphones in daily life, smartphone dependency has become a global problem, especially among adolescents. Existing research studies have supported the association between smartphone dependency and the mental health of Chinese rural adolescents, but the underlying mechanism is still unclear.

**Objective:**

The present study used a survey to test whether smartphone dependency may be associated with mental health in Chinese rural adolescents. The mediating role of cognitive failure and parent–child relationship was also examined.

**Materials and methods:**

In total, 941 adolescents (45.91% male; mean age = 14.05, SD = 1.04) in rural areas of mainland China were recruited to complete four scales, including the Mobile Phone Dependence Scale (MPDS), Cognitive Failures Questionnaire (CFQ), Family Adaption and Cohesion Evaluation Scales (FACES), and Mental Health of Middle School Students Scale.

**Results:**

The results showed that both cognitive failure and parent–child relationship acted as mediators in the effect of smartphone dependency on mental health among Chinese rural adolescents, and smartphone dependency also affected parent–child relationship by influencing cognitive failure, thus affecting mental health among Chinese rural adolescents indirectly.

**Conclusion:**

The present study suggests that improving parent–child relationships and reducing cognitive failure can reduce the impact of smartphone dependency on the mental health of Chinese rural adolescents.

## Introduction

With rapid social development, scientific and technological products, of which smartphones are the most popular and convenient, have become an important part of human daily life. In the meantime, smartphone dependency or smartphone addiction, defined as excessive or forced use of smartphones, has become one of the global issues, especially among adolescents (Yang et al., 2019; Zhen et al., 2020). According to the 50th Statistical Report on the Development of the Internet in China, as of June 2022, there were ~1.027 billion smartphone users in China, of which over 18% were adolescents under the age of 19 years (CNNIC, 2022). Due to the differences in China's socio-economic development between urban and rural areas, a large number of rural laborer flow into cities. The long-term weakened parent–child relationship and communication, as well as the lack of supervision and effective instruction from parents, may lead to more problematic behaviors among rural adolescents, such as substance abuse (Wang and Mesman, 2015) and smartphone dependency (Li and Hao, 2019). Furthermore, rural adolescents make up 48.76% of the total number of Chinese adolescents (National Bureau of Statistics of China, 2011). Therefore, attention must be paid to the smartphone dependency and mental health of Chinese rural adolescents.

### Smartphone dependency and mental health

Smartphone dependency, as the behavioral addiction to smartphones, has also been termed as mobile phone dependence, problematic smartphone use, and smartphone addiction (Goswami and Singh, 2016; Roser et al., 2016; Kil et al., 2021). These terms can be used interchangeably in some studies while may be conceptualized in different ways in others.

According to previous studies, smartphone dependency has been found to be associated with individuals' problems in mental health, such as anxiety and depression (Chen et al., 2017; Zhang et al., 2020). A recent literature review of 290 studies has reported that problematic smartphone use (dependency) is correlated with several negative outcomes in mental health, such as depression, stress, anxiety, and loneliness (Thomée, 2018). Such associations were also significant among Chinese adolescents (Li and Hao, 2019; Yang et al., 2019; Zhen et al., 2020; Lian et al., 2021; Wei et al., 2022).

The relation between smartphone dependency and mental health has been found not only in cross-sectional studies but also in longitudinal studies. Jun (2016) analyzed the 3-year longitudinal data of 1,877 Korean adolescents and found a two-way causal relationship between smartphone addiction and depressive symptoms over the past 3 years, based on the cross-lagged regression analysis. Another recent study showed that smartphone dependency in the 1st year predicted poor mental health in the 3rd year significantly among Chinese undergraduates (Zhang et al., 2020). However, more research studies are needed to draw effective conclusions about the correlation mechanism and causal direction between smartphone dependency and adverse mental health outcomes.

### Parent–child relationship and mental health

Parent–child relationship, as a lasting bond between caregivers and offspring (Cox and Paley, 2003), is the most fundamental and principal relationship in the family, which affects the mental health of children and adolescents. Theoretically, the social convoy model (Dohrenwend and Snell Dohrenwend, 1970) can illustrate the relation between parent–child relationship and mental health. In this model, the social relations around an individual can be separated into three concentric circles based on their sentimental proximity, in which the inner circle represents the closest relationship with the individual, often including close family members. Based on this model, parent–child relationship, as the most intimate social relationship among children and adolescents, has its own major influence on the mental health of children and adolescents.

An empirical research study further confirmed this influence of parent–child relationship on the mental health of children and adolescents (Morgan et al., 2012; Singh, 2014; Wu and Lee, 2020). For instance, Min et al. (2016) believed that parent–child relationship has different characteristics at different stages from individual childhood to adolescence, and different aspects of it have different effects on the mental health of children and adolescents, including parent–child attachment, parent–child conflict, and parent–adolescent cohesion. Positive parent–child relationship, as an important protective factor, helps to improve the mental health of children and adolescents (Min et al., 2016). However, negative parent–child relationships from stepparent families, single-parent families, and single-parent families with other relatives would adversely affect the mental health of children and adolescents (Barrett and Turner, 2005). Xia and Qian (2001) have found that many psychosomatic symptoms and other negative mental health outcomes of adolescents were significantly associated with parents' dismissal and rejection, punishment tendency, and lower levels of parental enthusiastic warmth and perception.

According to the displacement hypothesis, addiction to smartphones and other media can reduce face-to-face interactions with others in real life and lead to the decline of interpersonal relationships (Kuss and Griffiths, 2011; Chen et al., 2016). As an important component of adolescent interpersonal relationships, the parent–child relationship may worsen due to issues with adolescent smartphone use. Many studies have shown that problematic smartphone use may damage the quality of the parent–child relationship (Sahu et al., 2019; Kong et al., 2021). Empirical studies have revealed that negative parent–child relationships can positively predict the occurrence of depression and other mental health issues in adolescents (Yang et al., 2017; Wu and Lee, 2020).

### Cognitive failure and mental health

Cognitive failure is a cognitively based error that occurs during a process where a person typically successfully performs a task (Broadbent et al., 1982). The Cognitive Failures Questionnaire (CFQ) developed by Broadbent et al. (1982) is specifically designed to assess examples of cognitive errors in daily attention, perception, memory, and motor functions. Previous studies have found significant correlations between cognitive failure and psychological distress, such as somatic symptoms, anxiety, and depression (Brian, 1989; Houston and Allt, 1997).

Wallace and Chen (2005) proposed that it was worth examining the role of anxiety and stress in cognitive failure. They pointed out that stress and cognitive dysfunction were the main causes of accidents. Ohman et al. (2007) emphasized a problem where patients who perceive chronic stress also report cognitive dysfunction. In particular, they found that this dysfunction is a selective defect that mainly affects episode memory and distraction.

### The role of cognitive failure and parent–child relationship

According to the immersion theory, smartphone dependency will make the dependent pay too much attention to smartphone information, resulting in the disappearance of their self-awareness and sense of time (Csikszentmihaly, 1990). It will damage individuals' functions in both physiology and psychology, such as decreasing cognitive resources for attention, debasing working memory ability, and shortening the duration of sustained attention (Jonathan et al., 2007), which leads to cognitive failure.

Kahneman's (1973) cognitive resource theory holds that individual cognitive resources are limited. Because smartphones have consumed most of their psychological resources, smartphone addicts are prone to errors and cognitive failure in social interaction, which affects individual interpersonal relationships (Hu et al., 2021). Mathews et al. (1990) conducted a factor analysis and found cognitive failure associated with social interaction. Although there is a lack of relevant research, we can still infer that parent–child relationships, as the basis of interpersonal relationships, will also be affected by cognitive failure.

## Goal of the study

**Figure 1:**
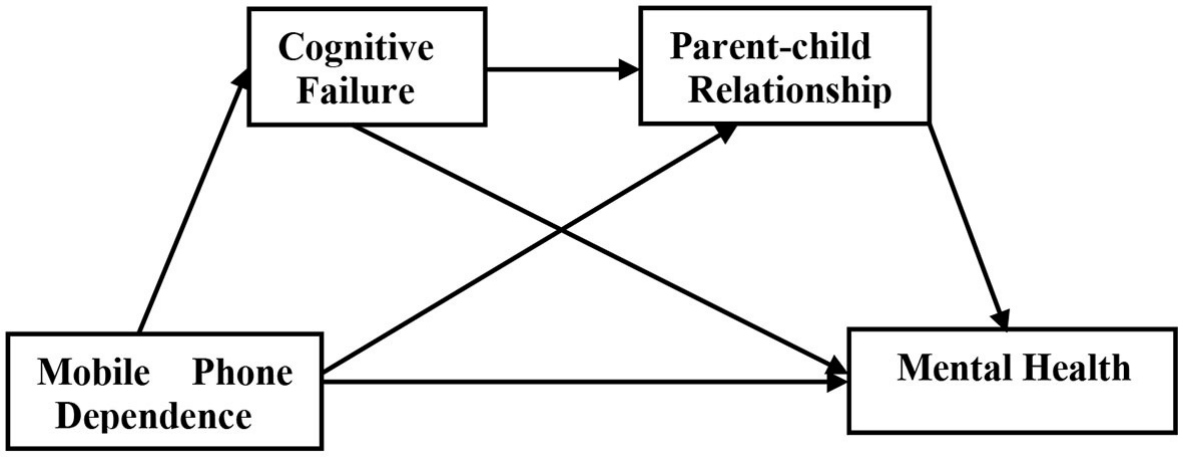
Proposed mediation model.

We put forward a serial mediation model (Figure 1) to test the mediating role of cognitive failure and parent–child relationship in the correlation between smartphone dependency and mental health. To be specific, we examined four hypotheses as follows:

Hypothesis 1: Smartphone dependency is directly correlated with Chinese rural adolescents' mental health.
Hypothesis 2: Smartphone dependency is indirectly correlated with Chinese rural adolescents' mental health via cognitive failure.
Hypothesis 3: Smartphone dependency is indirectly correlated with Chinese rural adolescents' mental health via parent–child relationship.
Hypothesis 4: Smartphone dependency is indirectly correlated with Chinese rural adolescents' mental health by cognitive failure and then parent–child relationship.

## Methods

### Participants

Through a convenient sampling method, 941 middle school students (45.91% male) with experience using smartphones were recruited for the study. We used the power analysis with G^*^Power to test the final sample size of 941 and three predictors as the baseline to further examine whether our valid sample and conclusions were representative and appropriate (Faul and Erdfelder, 1992). The results of *post-hoc* power analysis revealed that at the small effect size (*f* 2 = 0.05) for *R* square change, the power to detect the obtained effect for the whole regression in the prediction of mental health was 1.00, which was above the value of 0.8 recommended by previous research studies (Cohen, 1992; Fritz and MacKinnon, 2007). Hence, we can deduce that our final valid sample of 941 has enough power to detect small effects, and our conclusions based on this sample are appropriate and representative.

All participants came from two junior middle schools in rural areas of Hainan Province in China. The average age of participants was 14.05 years (SD = 1.04), with an age range of 12–17 years. Among them, 373 students (39.64%) were in seventh grade, 243 students (25.82%) were in eighth grade, and 325 students (34.54%) were in ninth grade.

### Measures

#### Smartphone dependency

Smartphone dependency was measured by the Mobile Phone Dependence Scale (MPDS) originally developed by Walsh et al. (2008). In the present study, the Chinese version revised by Wang (2011) included 16 items of three subscales: compulsion (e.g., “I often feel my mobile phone ringing or shaking”), withdrawal (e.g., “if I forget to bring my mobile phone, I will feel restless”), and prominence (e.g., “I often forget what to do because I play my mobile phone”). Cronbach's alpha reliability coefficient of the MPDS in the study was 0.90. Participants rated each item from 1 (strongly inconsistent) to 5 (strongly consistent) on the Likert 5-point scale.

#### Mental health

Mental health was measured by the Mental Health of Middle School Students Scale developed by Wang et al. (1997) which included 60 items of 10 subscales: obsession, paranoia, hostility, interpersonal sensitivity, depression, anxiety, learning pressure, maladjustment, emotional instability, and psychological imbalance. There are six items in each subscale. Participants rated each item from 1 (never) to 5 (seriousness) on the Likert 5-point scale. The higher the score, the more serious the individual's mental health problems are. Cronbach's alpha reliability coefficient of the mental health scale in the study was 0.98.

#### Parent–child relationship

Parent–child relationship was measured by the Family Adaption and Cohesion Evaluation Scales (FACES) founded by Olson et al. (1979) which included two subscales of father–child relationship and mother–child relationship, and each subscale had 10 items of the same measurement contents (e.g., “my father/mother and I support each other in times of difficulty” or “my father/mother and I like to spend leisure time together”). Participants rated each item from 1 (never) to 5(always) on the Likert 5-point scale. The higher the score, the better the individual's parent–child relationship is. Cronbach's alpha reliability coefficient of the parent–child relationship in the study was 0.84.

#### Cognitive failure

Cognitive failure was measured by the Cognitive Failures Questionnaire (CFQ) originally developed by Broadbent et al. (1982). In the present study, the Chinese version revised by Zhou et al. (2016) included 25 items of three subscales: attention failure (e.g., “When you do other things, you don't hear others call you”), memory failure (e.g., “You often forget why you walk from room to room”), and motor failure (e.g., “You lose your temper and often regret it” or “You often bump into others”). Cronbach's alpha reliability coefficient of the CFQ in the study was 0.93. Participants rated each item from 1 (strongly inconsistent) to 5(strongly consistent) on the Likert 5-point scale.

### Procedure

We informed participants of the requirements of this survey through standard instructions and asked them to fill out questionnaires for 20 min in class on a voluntary basis. After the Ethics Committee of Hainan Normal University approved the study, online signed consent forms were collected.

### Data analysis

We use the social science statistical software package (SPSS, version 26.0) for data analysis and processing. We first conducted descriptive statistics on demographic variables and all research variables and then performed correlation analysis. Since the distribution patterns of research variables were normal or approximately normal and the sample size of this study was large, the Pearson correlation coefficient was used to indicate the degree of correlation between continuous variables. Since gender is a dichotomous variable (male = 1, female = 0), the point biserial correlation analysis should be performed between gender and continuous variables. Because the point biserial correlation coefficient is the same as Pearson's correlation coefficient here, Pearson's correlation analysis was used in this study to test the correlation between variables. Finally, we used SPSS macro PROCESS Model 6 (with smartphone dependency as the independent variable, mental health as the outcome variable, cognitive failure and parent–child relationship as the mediators, and age and gender as the covariates) to conduct a bootstrap analysis on 5,000 resamples to test the serial mediation model and calculate the 95% CIs.

## Results

### Common method variance analysis

Before testing the hypothesis, we evaluated the common method variance (CMV) by performing Harman's one-factor test. According to Podsakoff and Organ (1986), if a common factor accounts for more than 40% of the total variance, it indicates that there is a common method variance. In this study, the EFA results showed that the eigenvalue of 17 factors exceeded 1, and the first factor accounted for 30.46% of the total variance, indicating that the problem of commonly used method variance in this study was not serious.

### Descriptive and Pearson's correlation

The descriptive statistics and Pearson's correlations for all of the assessed variables are presented in Table 1. Specifically, mobile phone dependence was positively associated with mental health (*r* = 0.49, *p* < 0.001) and cognitive failure (*r* = 0.56, *p* < 0.001). Similarly, positive relationships were also observed between mental health and cognitive failure (*r* = 0.68, *p* < 0.001). However, mobile phone dependence (*r* = −0.23, *p* < 0.001), cognitive failure (*r* = −0.24, *p* < 0.001), and mental health (*r* = −0.35, *p* < 0.001) were negatively correlated with parent–child relationship.

**Table 1:** Descriptive statistics and Pearson's correlations between the study variables.

**Variables**	***M***	***SD***	**1**	**2**	**3**	**4**	**5**	**6**
1 Gender	0.46	0.50	—					
2 Age	14.07	1.17	−0.03	—				
3 Mobile phone dependence	2.28	0.72	−0.07^*^	0.04	—			
4 Cognitive failure	2.37	0.63	−0.19^***^	0.10^**^	0.56^***^	—		
5 Parent–child relationship	65.43	11.96	0.12^***^	−0.07^*^	−0.23^***^	−0.24^***^	—	
6 Mental health	2.14	0.78	−0.19^***^	0.14^***^	0.49^***^	0.68^***^	−0.35^***^	—

* p < 0.05.

** p < 0.01.

*** p < 0.001.

Gender: male = 1, female = 0.

The two samples divided by gender showed homogeneity of variance (ps > 0.05) on all variables except the mental health variable, and the level of significance associated with gender was consistent with the level of significance of the independent sample *t*-test (Gravetter et al., 2020). Since gender and age were significantly correlated with research variables, they were used as control variables in subsequent regression analyses.

### Testing for a serial mediation model

**Figure 2:**
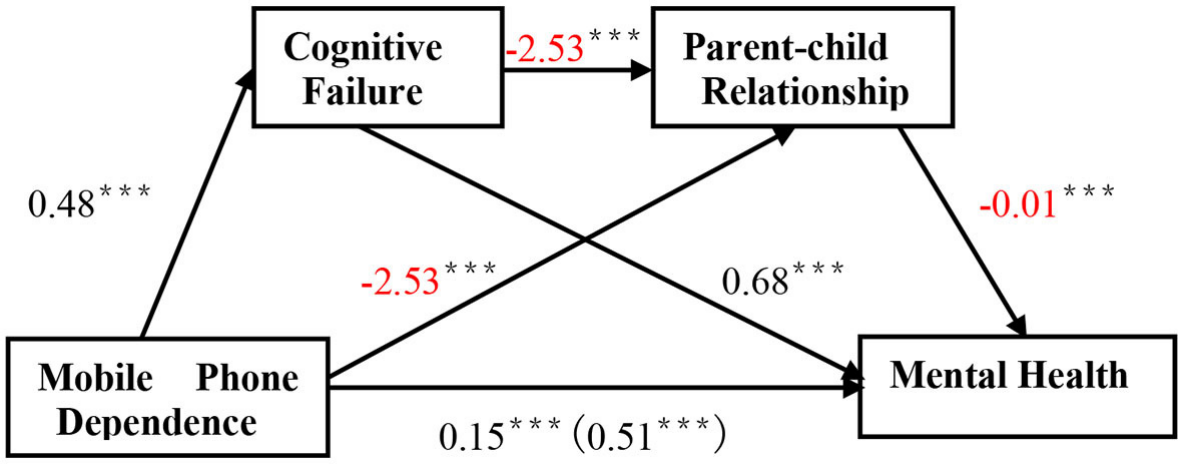
Serial mediation model shows effects of mobile phone dependence, cognitive failure, and parent–child relationship on mental health. *p < 0.05, **p < 0.01, ***p < 0.001.

We tested a serial mediation model, which consisted of three indirect effects, as follows: (1) smartphone dependency strengthens mental health via cognitive failure, (2) smartphone dependency strengthens mental health via parent–child relationship, and (3) smartphone dependency strengthens mental health via cognitive failure and then parent–child relationship (Figure 2).

After controlling for the effects of age and gender, the results presented a positive effect of smartphone dependency on cognitive failure (*B* = 0.48, *t* = 20.47, *p* < 0.001) but a negative effect of smartphone dependency on parent–child relationship (*B* = −2.53, *t* = −4.03, *p* < 0.001). There was also a negative relationship between cognitive failure and parent–child relationship (*B* = −2.53, *t* = −3.47, *p* < 0.001). Moreover, both cognitive failure and parent–child relationship significantly predicted mental health (*B* = 0.68, *t* = 19.54, *p* < 0.001 for cognitive failure and *B* = −0.01, *t* = −7.34, *p* < 0.001 for parent–child relationship). The total effect of smartphone dependency on mental health was statistically significant (*B* = 0.51, *t* = 17.03, *p* < 0.001). The direct effect of smartphone dependency on mental health was also significant, even after controlling for the effects of cognitive failure, parent–child relationship, age, and gender (*B* = 0.15, *t* = 4.96, *p* < 0.001).

In addition, the indirect effect of smartphone dependency on mental health through cognitive failure was significant [*B* = 0.323, SE = 0.027, 95% CI (0.272, 0.379)]. The mediation effect (smartphone dependency → cognitive failure → łł mental health) accounted for 63.27% of the total effect. Parent–child relationship mediated the relationship between smartphone dependency and mental health [*B* = 0.029, SE = 0.009, 95% CI (0.013, 0.047)]. The mediation effect (smartphone dependency → parent–child relationship → mental health) accounted for 5.53% of the total effect. Finally, the indirect effect of smartphone dependency on mental health through cognitive failure and then parent–child relationship (i.e., a serial mediating effect) was also found [*B* = 0.014, SE = 0.005, 95% CI (0.006, 0.024)]. The mediation effect (smartphone dependency → cognitive failure → parent–child relationship → mental health) accounted for 2.67% of the total effect. The direct and indirect effects of cognitive failure and parent–child relationship on the relationship between smartphone dependency and mental health are presented in Table 2.

**Table 2:** Direct, indirect, and total effects of mobile phone dependence on mental health.

**Model pathways**	**Estimated effect (β)**	**95% CI**	
		**Lower**	**Upper**
**Direct effect**			
MPD → MH	0.148^***^	0.000	0.089
**Indirect effect**			
MPD → CF → MH	0.323^***^	0.272	0.379
MPD → PCR → MH	0.029^***^	0.013	0.047
MPD → CF → PCR → MH	0.014^***^	0.006	0.024
Total effect	0.510^***^	0.451	0.569

*** p < 0.001.

MPD, mobile phone dependence; MH, mental health; CF, cognitive failure; PCR, parent–child relationship.

Due to the statistical significance of these three indirect effects (consisting of the mediating effect of cognitive failure in the relation between smartphone dependency and mental health, the mediating effect of parent–child relationship in the relationship between smartphone dependency and mental health, and the series of mediating effects of cognitive failure and parent–child relationship in the relation between smartphone dependency and mental health), we examined whether there were significant differences in the mediating effects of these effects. There was no significant difference between the mediating effects of parent–child relationship and the series of mediating effects of cognitive failure and parent–child relationship [*B* = 0.014, SE = 0.010, 95% CI (−0.005, 0.033)]. Nevertheless, the mediation effect of cognitive failure was stronger than the mediation effect of parent–child relationship [*B* = 0.272, SE = 0.026, 95% CI (0.223, 0.325)] and the series of mediation effects of cognitive failure and parent–child relationship [*B* = 0.286, SE = 0.024, 95% CI (0.240, 0.335)].

## Discussion

This study investigated the ways by which smartphone dependency, cognitive failure, and parent–child relationship affected the mental health of Chinese rural adolescents. The results showed that smartphone dependency could affect the mental health of Chinese rural adolescents directly, which corresponded to the first hypothesis in our study and the findings of other earlier studies (Thomée, 2018; Li and Hao, 2019; Yang et al., 2019; Zhen et al., 2020; Lian et al., 2021). In addition, our findings support the second and third hypotheses that cognitive failure or parent–child relationship could conduct as the internal mechanism that might give a partial explanation for the link between smartphone dependency and mental health in Chinese rural adolescents.

According to the cognitive resource theory (Kahneman, 1973), smartphone dependency will occupy more cognitive resources when using mobile phones. Such continuous concentration can easily cause the decline of working memory capacity and attention control ability, and the continuous use of smartphones will make the dependent focus on virtual things in the network so as to reduce the unintentional attention to real stimuli in the real environment, resulting in cognitive failure (Hadlington, 2015). Cognitive failure is closely related to individual mental health, which is basically consistent with previous studies (Brian, 1989; Houston and Allt, 1997).

In accordance with the time displacement hypothesis (Kraut et al., 1998), media use (dependence) leads to the reduction of social interaction between individuals and their families, and the quality of the relationship decreases, which affects their mental health. Therefore, we confirmed a mediation effect of parent–child relationship on the correlation between cognitive failure and mental health and inferred the serial mediating model of smartphone dependency → cognitive failure → parent–child relationship → mental health, which has been affirmed by the study, that is to say, it has been found that cognitive failure and parent–child relationship had a share in some of the variances and made a significant contribution to explaining the impact of smartphone dependency on the mental health of Chinese rural adolescents. The indirect effect of cognitive failure on mental health was mediated by parent–child relationships.

This study reveals the important role of smartphone dependency, cognitive failure, and parent–child relationship in Chinese rural adolescents' mental health and explores the serial mediating role of cognitive failure and parent–child relationship. The advantage of this study is that it can help broaden the understanding of individual and family environmental variables that may affect mental health. This study covers an important area and offers some new information. The results of the study indicate the importance of cognitive failure, which has important implications for repairing the impairments in parent–child relationship and mental health of rural adolescents in China. Thus, cognitive-behavioral intervention based on cognitive failure might help to alleviate the impact of smartphone dependency on adolescents' mental health.

## Limitations and future recommendations

This study has several limitations. First, we could not infer the causal relationship between variables accurately for the cross-sectional study, and longitudinal and experimental designs shall be carried out in future. Second, the parent–child relationship was only evaluated based on adolescents' self-reports, and data from parents could be adopted in future research. Third, the results could not be popularized in other samples because our participants were recruited from two secondary schools in the same region. In future, replication studies should be carried out on more adolescent samples from different rural areas in China.

## Conclusion

In summary, our findings show a positive relationship between smartphone dependency and the mental health of Chinese rural adolescents, and cognitive failure and parent–child relationship play a mediating role in smartphone dependency and mental health of Chinese rural adolescents. The present study expanded our understanding of the mechanism underlying smartphone dependency and mental health. The serial mediation effects suggest that improving parent–child relationship and reducing cognitive failure can reduce the impact of smartphone dependency on the mental health of Chinese rural adolescents.

## Data availability statement

The raw data supporting the conclusions of this article will be made available by the authors, without undue reservation.

## Ethics statement

The studies involving humans were approved by the Ethics Committee of Hainan Normal University. The studies were conducted in accordance with the local legislation and institutional requirements. Written informed consent for participation in this study was provided by the participants' legal guardians/next of kin.

## Author contributions

LC designed the study, carried out the experiment, analyzed the data, and wrote the manuscript.
